# Treatment of Tubercular Cystitis

**Published:** 1900-06

**Authors:** 


### TREATMENT OF TUBERCULAR CYSTITIS.

Ramon Guiteras (*Medical News*, April 7, 1900,) says:

Numerous remedies have been recommended by different authorities for the treatment of this form of cystitis, and naturally every practitioner who encounters this rebellious trouble grasps at anything that offers the probability of a cure. Guyon at one time advocated the use of intravesical injections of bichloride of mercury, 1 to 10,000 and since then many have been following his advice, but such a solution will rarely cure this disease, while it usually produces an irritation that is almost unbearable.

Nitrate of silver and permanganate of potassium have the same effect. Boric acid and boroglycerine irritate less, but do not seem to possess the power to ameliorate the disease. Recently iodoform injections have been advocated, and the procedure would seem to be founded on a logical basis. Three or four ounces of a 5 per cent. solution of iodoform in liquid vaseline are injected into the bladder every two or three days, the patient being instructed to watch the stream when he urinates and stop the flow just as soon as the oil appears. This forms a permanent iodoform dressing of the bladder wall, and in the hands of some of the French surgeons is said to have met with gratifying results.

Personally, I have had better results with borolyptol in this class of cases than with any other remedy which I have employed. This seems to have a powerful germicidal effect, while the fact that it does not irritate the bladder renders it pleasant to the patient. It is used in the strength of from 1 in 8 to 1 in 16 in irrigations by the hydrostatic method. After a few irrigations at the office, the patient will be able to use it every night at home. I have one patient now under observation who suffered for a number of years from a most aggravating frequency of urination accompanied by pain, dependent upon a tubercular cystitis. Under this treatment the urine has cleared up, the tubercle bacilli have disappeared and the patient can hold his urine from seven to nine hours at night and from four to six hours during the day.

Internally, in connection with any local treatment, an antispasmodic and an internal antiseptic should be used as a palliative measure; it is wonderful how much relief may be given to the patient by this means, even although pus remains in the urine and the tubercle bacilli still be found. One patient has been coming to me for three months who was entirely relieved of this disagreeable subjective symptom by a mixture containing ten minims of the tincture of belladonna, fifteen grains of benzoate of soda, and oil of gaultheria up to one drachm, t. i. d., although not until he was put on the borolyptol irrigation did the pus and tubercle bacilli in the urine diminish to any marked degree. The effect of the palliative internal medication is worthy of notice, in view of the fact that he had suffered for fourteen years, and had been under the care of many different physicians without relief, having most probably been overtreated by too much instrumentation and too frequent or too irritating irrigations.

